# Optical trapping and fluorescence control with vectorial structured light

**DOI:** 10.1038/s41598-022-21224-1

**Published:** 2022-10-21

**Authors:** Ané Kritzinger, Andrew Forbes, Patricia B. C. Forbes

**Affiliations:** 1 Department of Chemistry, University of Pretoria, Pretoria, South Africa; 2 School of Physics, University of the Witwatersrand, Johannesburg, South Africa

**Keywords:** Chemistry, Physics, Optics and photonics, Applied optics, Optical materials and structures, Optical techniques

## Abstract

Here we functionalized micro-scaled polymer beads with nano-scaled quantum dots and demonstrate optical trapping and tweezing, with in-situ fluorescence measurement, in an all-digital all-optical configuration. We outline the chemistry required to facilitate this, from deactivating the optical trapping environment to size, adhesion and agglomeration control. We introduce a novel holographic optical trapping set-up that leverages on vectorially structured light, allowing for the delivery of tuneable forms of light from purely scalar to purely vector, including propagation invariant flat-top beams for uniform illumination and tailored intensity gradient landscapes. Finally, we show how this has the potential to quench bleaching in a single wavelength trap by linear (spatial mode) rather than non-linear effects, advancing the nascent field of optics for chemistry.

## Introduction

Optical trapping or tweezing describes the manipulation of nano- to micro-sized particles through momentum transfer from tightly focused light. Optical tweezing was first demonstrated by Arthur Ashkin in 1970 with a Gaussian beam^1^ and half a century later this beam still dominates optical trapping experiments^2^. However, the employment of structured light^3^ (by varying the intensity, phase and polarization of light) in optical tweezers has made it possible to not only trap but to move, rotate and direct particles. These structured light traps are a well-established technique today^4,5^ and since most structured beams are created by means of a hologram, they have been dubbed holographic optical tweezers (HOTs)^6,7^.

With HOTs, an array of traps can be created to trap multiple particles simultaneously while being able to dynamically change this array pattern, allowing for highly controlled manipulation of particles^8–11^. Structured beams that reconstruct themselves after being distorted by a trapped particle (Bessel beams) have allowed for trapping in multiple planes^12^, whereas the far-field Bessel beam can be used as an optical shield assisting with trapping in crowded environments^13^. Structured light beams have not only been shown to enhance the trap strength^14^ but Airy beams, for example, can guide a particle along a certain trajectory enabling selective removal of particles in a sample^15,16^; with petal beams it is possible to trap particles with different refractive indices simultaneously^17^ and frozen waves can increase the stability and 3D control of the trap^18^. The fact that light carries linear momentum is well-known and is the reason why light can trap particles, however, light can also carry orbital angular momentum (OAM), like Laguerre-Gaussian (LG) beams. By employing these OAM carrying beams, optical tweezers also gain rotational control of particles^19–22^.

So far HOT research has focused mainly on structured beams modulated in amplitude and phase—these are called scalar beams. On the other hand, vector beams are structured in polarization as well, meaning they have a spatially varying polarization pattern. Trapping with vector beams is the most recent avenue of structured light explored in optical tweezers and has already proven beneficial to the trapping community^4,5,23^. The radially polarized vector beam, for example, is famous for achieving the smallest spot size when tightly focused^24,25^, this property has been used to create stronger axial optical traps^26,27^. The first vectorial HOT was demonstrated by Bhebhe *et al.*^28^, which enabled optical trapping with a dynamic array of vector and/or scalar beams.

Using structured light in optical tweezers has made it a powerful technique, furthermore, combining this tool with fluorescence spectroscopy made it possible to not only exert forces on a particle but also to observe chemical and structural changes of molecules within the trap. For this reason, optical tweezers combined with single molecule fluorescence is an invaluable and pioneering tool in biology research today^29–31^. The integration of fluorescence microscopy into optical tweezers is however not trivial, since the trapping light has an intensity up to six orders of magnitude higher than that of excitation light used in fluorescence experiments^32^. The high intensity trapping light result in photobleaching of the fluorophores, which is an irreversible process whereby fluorophores become non-fluorescent^33^. Although not fully understood, photobleaching usually occurs when already excited electrons continue to absorb photons and the resulting dissociation then leads to permanent loss of fluorescence signal^34^. The most popular solution to minimize photobleaching in optical tweezers is to use two different sources—one non-resonant high intensity laser for trapping and a resonant lower intensity source for excitation^34,35^. These two sources are then separated either in space^36–38^ or in time^39,40^. Very little research has investigated the possibility of using structured light to assist with integrating fluorescence spectroscopy in optical tweezers, and none to date exploits the vectorial nature of structured light, groups have only investigated using a vortex or ‘doughnut’ trapping beam to reduce photobleaching^41–43^. Recently, Zhang and Milstein showed that the photobleaching lifetime of an organic dye positioned 1 $\upmu \text{m}$ below the trap centre can be extended by trapping with a vortex beam, while still using separate trapping and excitation sources^43^.

Here we demonstrate optical trapping and tweezing with vectorial light for the control of fluorescent particles. The fluorescent particles used in this study are semiconducting nanocrystals known as quantum dots (QDs). We first discuss in detail the chemistry to create QD probes, from QD synthesis to their coupling to micro-sized polymer beads for trapping. Our functionalized micro-scaled polymer beads with nano-scaled quantum dots are then introduced to a novel holographic optical trapping set-up that leverages on vectorially structured light, allowing us unprecedented control in tailoring the gradient forces and intensity profiles within the trap. We demonstrate this with the delivery of tuneable forms of light from purely scalar to purely vector. As an illustrative example of the power of our set-up and approach, we create a propagation invariant vector flat-top beam and show its potential to reduce photobleaching in a single wavelength trap by linear (spatial mode) rather than non-linear effects, with exciting prospects in exploring structured light for chemistry.

## Results

**Figure 1:**
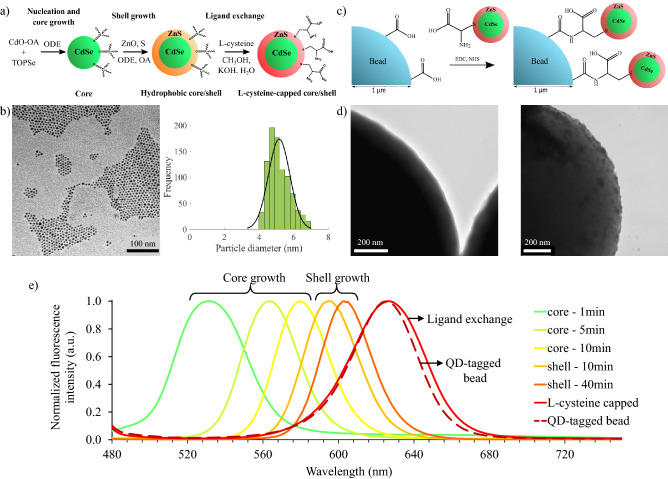
Synthesis steps and salient characterization of the QDs and QD-tagged beads. (**a**) L-cysteine capped CdSe/ZnS QD synthesis steps. (**b**) TEM image of CdSe/ZnS QDs with their corresponding size distribution; the average diameter was 5.2 ± 0.6 nm. (**c**) The coupling reaction of L-cysteine capped QDs to the surface of micro-sized beads through EDC/NHS chemistry. (**d**) TEM images of the surface of an uncoated polymer (left) and a QD-tagged bead (right), confirming the success of the coupling reaction. (**e**) Fluorescence emission of the QDs at different times throughout the synthesis.

CdSe/ZnS core/shell QDs were prepared using the hot-injection colloidal synthesis method followed by a ligand exchange reaction to functionalize the surface with L-cysteine. A schematic of the synthesis process followed is shown in Fig. 1a (see "Materials and methods" for detail). The TEM image of the hydrophobic QDs and their size distribution in Fig 1b show that the QDs had an average diameter of 5.2 ± 0.6 nm. To create fluorescence probes that can be trapped with the optical tweezer setup, these QDs were coupled to micro-sized polymer beads as shown in Fig. 1c. Well-known and widely used EDC/NHS chemistry was used to bond the QDs to the surface of the beads. The carboxyl groups present on the surface of the commercial polystyrene beads reacted with the primary amine group of the L-cysteine ligands on the QDs to form a covalent bond between the bead and the QD. TEM images of the surface of an uncoated commercial polymer bead and a QD-tagged bead are compared in Fig. 1d. The uncoated polymer bead had a smooth surface whereas the surface of the QD-tagged bead had a rough or ‘fuzzy’ appearance from the QD coating, confirming the success of the coupling reaction. The normalized fluorescence intensity of QDs at different times during the synthesis is shown in Fig. 1e. Longer growth time for the CdSe core and CdSe/ZnS core/shell QDs lead to larger particles (smaller bandgap) and therefore a red-shift in the fluorescence emission wavelength was observed. The ligand exchange reaction also caused a red-shift in the emission wavelength. After the coupling reaction, however, a slight blue shift was observed. In order to detect the fluorescence emission of the QD-tagged beads in the optical tweezer setup, the fluorescence light had to pass through a dichroic mirror that transmits light with wavelengths of 582-825 nm. To ensure most of the emission peak is longer than 582 nm, the QDs were grown to have a fluorescence peak maximum of 595 nm or longer.

**Figure 2:**
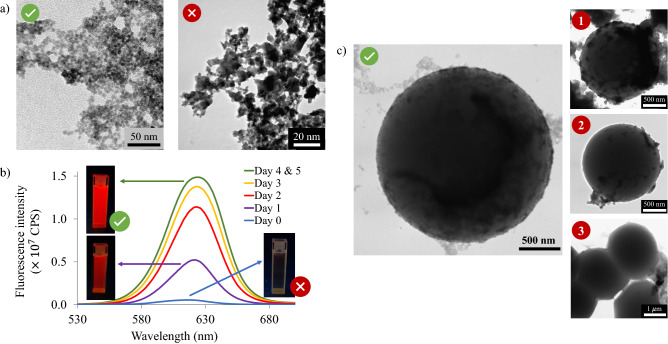
Optimisation steps when preparing the QD-tagged beads. (**a**) TEM images of purified (left) and crude (right) L-cysteine capped CdSe/ZnS QDs. (**b**) Fluorescence intensity recovery of L-cysteine capped CdSe/ZnS QDs over five days. The photo insets show the appearance of the sample under UV-light on certain days. (**c**) The TEM image on the left shows a QD-tagged bead uniformly coated with QDs, whereas insets 1–3 show QD-tagged beads when some synthesis steps were not carried out optimally. In inset 1 the excess EDC was not removed before adding the QDs, inset 2 shows the reaction done with magnetic stirring as opposed to ultra-sonication and inset 3 shows the deformation of the polymer beads due to the presence of acetone.

The importance of optimising some of the synthesis steps when preparing the QD-tagged beads are highlighted in Fig. 2. Firstly, the purification of the L-cysteine capped QDs can be time consuming, but is a crucial step in order to have a monodispersed and impurity free QD sample and consequently a uniform QD coating on the polymer beads. In Fig. 2a, the appearance of purified L-cysteine capped QDs is shown on the left, here individual QDs are visible, whereas a TEM image of crude L-cysteine capped CdSe/ZnS QDs before purification is shown on the right. The crude sample is clumped together with many impurities between the QDs so that no individual QD can be seen. The large surface area of QDs provides organic impurities with much space for attachment, therefore the need for rigorous purification.

After purification and drying of the L-cysteine-capped CdSe/ZnS QDs, their fluorescence intensity was quenched. However, after some time the fluorescence intensity recovered when they were redispersed in water as shown in Fig. 2b. This figure shows the recovery of the fluorescence over a period of 5 days; the first measurement was taken directly after the purified QDs were redisperesd in water. This quenching phenomenon can be explained by the research published by Noh et al.^44^. In this work they showed that the fluorescence of water-soluble CdSe QDs quenched when the QDs formed aggregates. Similarly, we know that the L-cysteine capped QDs exhibit hydrogen bonding and tend to clump together. Thus the L-cysteine QDs possibly formed aggregates when concentrated which resulted in the fluorescence quenching. When they were redispersed in water the aggregates dispersed and the fluorescence emission recovered. To ensure the fluorescence signal from the QDs was recovered and stable, the QDs were stored in deionized water for several days before performing the coupling reaction.

On the left of Fig. 2b a TEM image of a QD-tagged bead with a uniform QD coating is shown, whereas insets 1-3 show QD-tagged beads when some synthesis steps were not carried out optimally. Inset 1 shows a QD-tagged bead when the excess EDC was not thoroughly removed before adding the QDs. The unreacted EDC activated not only the carboxyl groups on the beads but also the carboxyl groups of L-cysteine on the QDs. These activated QDs then reacted with each other to form large aggregates. Inset 2 shows the uneven QD coating when the coupling reaction was carried out with magnetic stirring. The amount of QDs on the surface of this bead ranged from almost nothing on the one side to large clumps on the other, however performing the reaction in an ultra-sonic bath greatly improved the uniformity of the QD coating (as shown on the left). Lastly, we would like to point out the importance of carefully studying the system under investigation before attempting a synthesis. Even though acetone proved to be an excellent solvent for purification of the QDs, in the QD-bead system this solvent caused the polymer beads to deform as shown in inset 3; only distilled water was thus used for purifying the QD-tagged beads.

### Vectorial holographic optical tweezer

**Figure 3:**
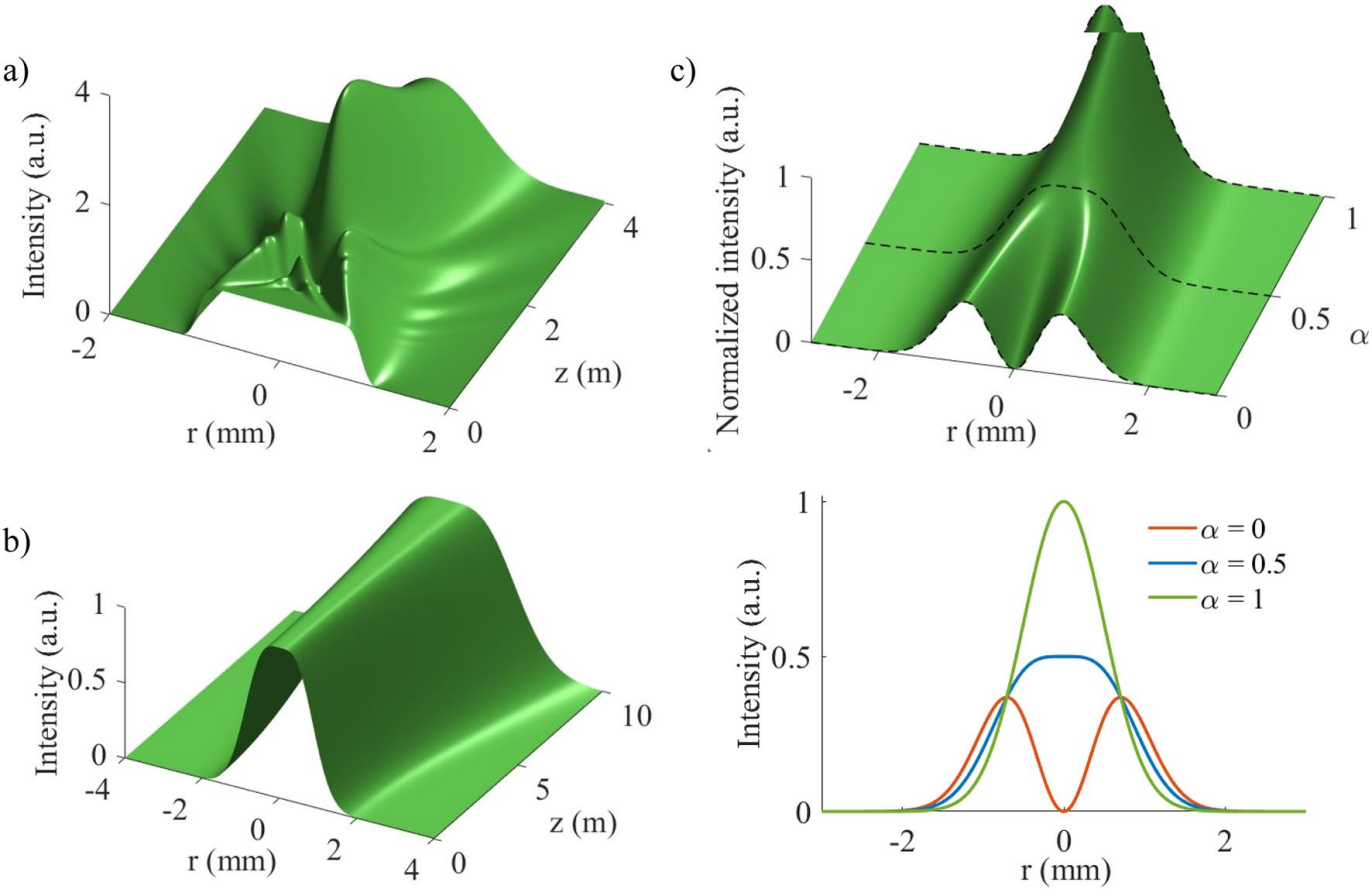
(**a**) Propagation of a scalar flat-top beam simulated using the flattened Gaussian beam approach. (**b**) Propagation of a vector flat-top beam showing the unchanging flat-top profile. The peak intensities were normalized to better visualize the propagation invariance of this beam. (**c**) Intensity profile as $\alpha$ changes showing the evolution from a vortex to a flat-top to a Gaussian beam. The profiles of the vector beam at critical $\alpha$ values are shown in the bottom panel with the vortex beam at $\alpha =0$, the vector flat-top beam at $\alpha =0.5$ and the Gaussian beam at $\alpha =1$.

Here we demonstrate a vectorial HOT by focusing on vector flat-top beams. This beam is beneficial for optical trapping of fluorescent particles since it provides uniform excitation illumination, it is a propagation invariant beam and has a steep intensity gradient (meaning it can produce a strong optical trap), while having a lower peak intensity to reduce photobleaching.

#### Vector flat-top beams

An ideal flat-top beam has a uniform intensity profile and falls to zero at the edges. However, in the laboratory only approximations to a flat-top beam can be created; some approximations include the super-Gaussian, flattened Gaussian and Fermi-Dirac beams^45,46^. The major drawback of all these approximations is that their intensity profile varies as they propagate, the flat-top intensity profile is only obtained at a certain plane after which the profile changes quite drastically as shown in Fig. 3a. The quick change in profile makes optical delivery of the flat-top profile at the tightly focused trapping plane extremely difficult. A beam that can simply be focused through the objective onto the sample and keep its profile is much more ideal—this is achieved by vector flat-top beams. Vector flat-top beams are propagation invariant since they are created by the superposition of two eigenmodes of free-space; the propagation of a vector flat-top beam is shown in Fig. 3b.

A vector flat-top beam is obtained through the (vector) addition of a Gaussian and a vortex beam^47^. The field is therefore given by

1 $$\begin{aligned} U_{FT} = \sqrt{\alpha } \text {LG}_0^0 {\hat{{\textbf {e}}}_{{\textbf {H}}}} +\sqrt{1-\alpha }\text {LG}_0^1{\hat{{\textbf {e}}}_{{\textbf {V}}}}\,, \end{aligned}$$

where $\hbox {LG}^l_{p}$ refers to the Laguerre-Gaussian (LG) modes with *p* the radial index and *l* the azimuthal index. $\hbox {LG}_0^0$ is thus the Gaussian beam and $\hbox {LG}_0^1$ the vortex beam. A vector beam is formed by the addition of orthogonal scalar fields with uniform polarization, here the Gaussian beam has horizontal polarization ${\hat{{\textbf {e}}}_{{\textbf {H}}}}$ and the vortex beam vertical polarization ${\hat{{\textbf {e}}}_{{\textbf {V}}}}$. In Eq. (1), a factor $\alpha$ was introduced to weigh the two scalar beams, meaning any field from a vortex when $\alpha =0$ to a Gaussian beam when $\alpha =1$ can be generated; with the vector flat-top at equal weighting of $\alpha =0.5$. The evolution of the vector beam as $\alpha$ changes is shown in Fig. 3c along with the beam profiles at the critical $\alpha$ values ($\alpha = 0, 0.5$ and 1). The $LG_p^l$ field takes the well-known form^48^

2 $$\begin{aligned} \begin{aligned} LG_p^l(r,\varphi ,z)&= \sqrt{\frac{2p!}{\pi (|l| + p)!}}\frac{1}{ w(z)}\left( \frac{\sqrt{2}r}{w(z)} \right) ^{|l|} L_p^{|l|}\left( \frac{2r^2}{w^2(z)}\right) \\&\times \exp [{i(|l| + 2p + 1)\psi (z)}]\exp [{il\varphi }] \\&\times \exp \left[ {-\frac{ikr^2}{2R(z)}}\right] \exp \left[ {-\frac{r^2}{w^2(z)}}\right] \,, \end{aligned} \end{aligned}$$

where $L_p^{|l|}$ is the associated Laguerre polynomial, *w*(*z*) $=w_0\sqrt{1 + \left( \frac{z}{z_R}\right) ^2}$, $w_0$ is the Gaussian beam radius, $z_R=\frac{\pi w_0^2}{\lambda }$ is the Rayleigh range, $R(z)=z\left( 1 + \left( \frac{z_R}{z}\right) ^2\right)$ is the radius of curvature and $\psi (z)=\text {arctan}\left( \frac{z}{z_R}\right)$ is the Gouy phase.

Here we (theoretically) compare the vector flat-top beam to a Gaussian beam to determine in what aspects flat-top beams are superior to Gaussian beams (and vice versa). The intensity of the vector beam can generally be written as the sum of the intensities of the Gaussian and vortex beam (with some polarization requirements)

3 $$\begin{aligned} I_{vector} = \alpha |\text {LG}_0^0|^2 + (1-\alpha )|\text {LG}_0^1|^2\,, \end{aligned}$$

where the vector flat-top intensity is obtained by setting $\alpha =0.5$ and the Gaussian intensity by $\alpha =1$.

The gradient force (or trap strength) of a beam is proportional to the intensity gradient of the beam, $F_{grad}=c\nabla I$^49–51^. Since only the relative forces of the vector flat-top and Gaussian beam are important, $c=1$ can be assumed such that

4 $$\begin{aligned} F_{grad} = \nabla I\,. \end{aligned}$$

Therefore, if the intensity profile of a beam is known, its gradient force can easily be calculated.

For the vector flat-top beam to be useful for trapping fluorescent particles, it must have a similar trap strength as a Gaussian beam but a lower peak intensity to possibly reduce photobleaching. To test when this is true, the average gradient force (${\bar{F}}_{grad}$) over the whole area of the beam, *A*, was considered

5 $$\begin{aligned} {\bar{F}}_{grad} = \frac{\int \! F_{grad} \, \mathrm {d}A}{\int \,\mathrm {d}A}\,. \end{aligned}$$

More relevant is the ratio of the average gradient force of the flat-top (FT) to the Gaussian (G) beam

6 $$\begin{aligned} \gamma = \frac{{\bar{F}}_{FT}}{{\bar{F}}_{G}}\,, \end{aligned}$$

so that at $\gamma = 1$ the average gradient force of the flat-top and Gaussian beams are equal and at $\gamma > 1$ the vector flat-top has a stronger trap strength.

**Figure 4:**
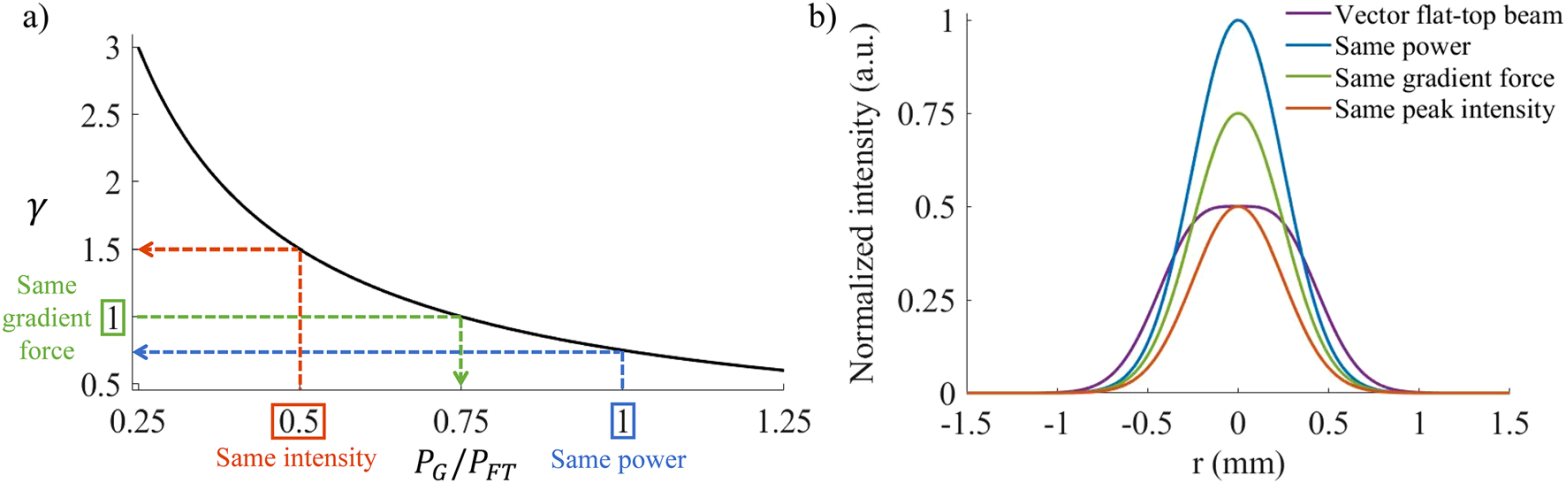
Comparing the gradient force of a Gaussian and vector flat-top beam. (**a**) The relationship between the relative gradient force and power of a Gaussian and vector flat-top beam. (**b**) The intensity profiles corresponding to a Gaussian beam having the same power, gradient force and intensity as a vector flat-top beam.

The ratio, $\gamma$, was calculated at different relative powers of the Gaussian and vector flat-top beams and plotted in Fig. 4a. This figure, therefore, shows how the relative gradient forces of the two beams change when adjusting the power. Three special cases are indicated on the graph with the corresponding intensity profiles shown in Fig. 4b: the vector flat-top beam along with Gaussian beams that have (1) the same power, (2) the same gradient force and (3) the same peak intensity as the flat-top are plotted. Firstly, in the instance when the power of the two beams are equal, $\gamma =0.75$, meaning the Gaussian beam is a stronger trap (blue line). This is expected, since even though the intensity gradient of the vector flat-top is steeper, the high intensity peak of the Gaussian beam negates this effect. Secondly, for the two beams to have the same average gradient force ($\gamma =1$), the Gaussian beam must have 75% of the power of the vector flat-top beam (green line). Lastly, when the two beams have the same peak intensity, that is when the power of the Gaussian is half that of the flat-top beam, then the flat-top beam trap is 1.5 times stronger than the Gaussian beam trap (orange line). Most relevant is the case when the two beams have the same gradient force; here it is clear that when the vector flat-top beam and the Gaussian beam have the same trap strength (or gradient force), the peak intensity of the vector flat-top is lower than the Gaussian beam. This means that the vector flat-top beam can be used to trap a particle with the same strength but with a lower peak intensity than the Gaussian beam.

#### Vector HOT setup

**Figure 5:**
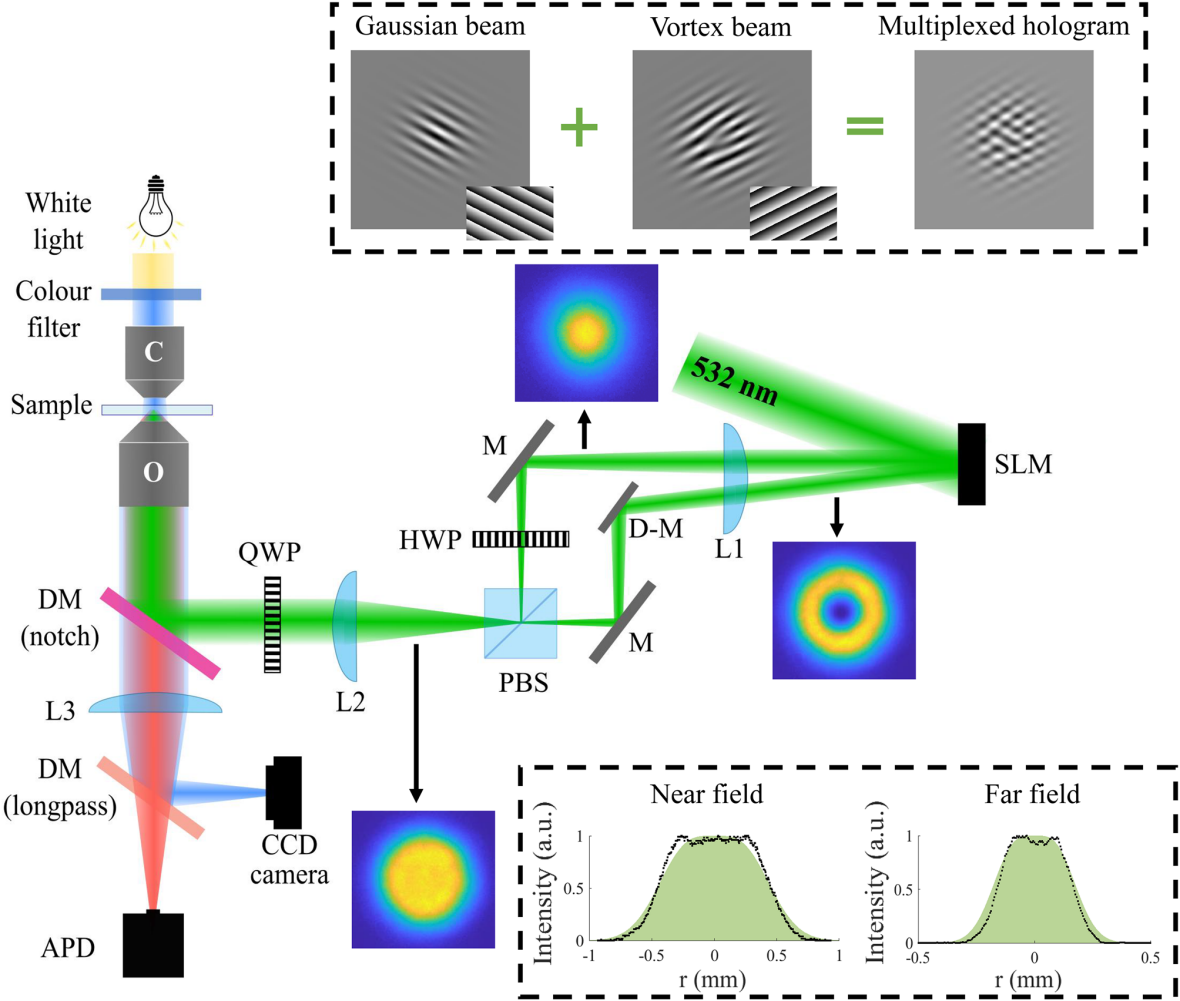
An illustration of the experimental setup of a vector holographic optical tweezer. The insets show the experimentally generated vortex and Gaussian beams that were combined to form the vector flat-top beam. The multiplexed hologram that was encoded on the SLM to generate a Gaussian and vortex beam with different propagation angles is shown in the top panel. The bottom panel shows the cross-section of an experimentally generated flat-top beam in the near and far field. Theoretical cross-sections are presented in green with the experimental data points in black.

The experimental setup of the vector holographic optical trap is illustrated in Fig. 5. An expanded and collimated Gaussian beam with a wavelength of $\lambda$ = 532 nm was used to illuminate the screen of a reflective spatial light modulator (SLM, Holoeye Pluto, Germany). To create a vector flat-top beam, a Gaussian ($\hbox {LG}^0_0$) and a vortex beam ($\hbox {LG}^1_0$) with different propagation angles were created with the SLM (the insets show the 2D intensity profiles of experimentally obtained beams). The multiplexed grey-scale hologram that was encoded on the SLM using complex amplitude modulation, is shown in the top panel, with the different gratings of the two beams as insets. The Gaussian and vortex beams were separated using a D-shaped mirror (D-M) in order to direct the beams to a polarizing beam splitter (PBS) where they were interferometrically combined. The unwanted zeroth and higher orders were removed by spatial filtering before the PBS. A half wave plate (HWP) was added in the path of one beam to change its polarization from horizontal to vertical, to allow for the superposition of orthogonal polarized beams. The vector flat-top beam was therefore obtained after the PBS; the bottom panel shows the cross-section of an experimentally obtained flat-top beam in the near and far field.

The reflection of light from a dichroic mirror (DM) is slightly different for horizontally and vertically polarized light. In order to ensure that the DM did not change the profile of the vector beam (given that its performance varies slightly for the orthogonal polarizations), a quarter wave plate (QWP) at $45^{\circ }$ was added in the path of the vector beam to change the polarization of each beam to circular, meaning the two beams making the vector light have the same ‘amount’ of vertical and horizontal polarization and the DM will have the same effect on both (the beam profiles with and without the QWP are shown in Supplementary Fig. S1). The 4f-system (lenses L1 and L2) was included to ensure the generated beam reached the back aperture of the objective lens. The high NA objective lens O focused the beam to create the optical trap in the plane of the sample.

The sample consisted of either 2 $\upmu \text{m}$ polystyrene beads (sample used for obtaining trap stiffness) or QD-tagged polystyrene beads (fluorescent sample) supported between a cover slip and microscope slide. Untreated glassware contains silanol groups (Si–OH), which make the surface of the glass hydrophilic, causing polar compounds to adsorb to the surface through hydrogen bonding. In this study, the polystyrene beads and the QD-tagged beads contained polar groups on their surfaces. Thus, due to these groups, the beads became immobilized on the surface of untreated glass which caused a problem when attempting to trap the particles. To solve this, the glassware (microscope slides and cover slips) was deactivated before assembling the samples. Deactivation of the glassware increased its hydrophobicity and prevented the unwanted adsorption of polar compounds. Deactivation was achieved by reacting the glassware with dimethyldichlorosilane (DMDCS).

An inverted microscope setup was implemented with a DM reflecting the laser light into the objective while letting the fluorescence and illumination light pass through. The same laser was used for both trapping and excitation. In order to simultaneously observe trapping and fluorescence emission from the sample, the imaging/detection system was set up such that blue light was used to illuminate the sample which was transmitted by the notch DM, reflected by the longpass DM and imaged to a CCD camera. The fluorescence emission (red beam) from the sample was transmitted by both DMs to be detected by an avalanche photodiode (APD, a single photon detector). This sensitive photon detector was necessary to detect the fluorescence coming from a single QD-tagged bead. Since the DMs are not 100% effective, extra color filters were inserted to ensure no light from the trapping laser reached the camera or APD.

Even though this study focused on creating vector flat-top beams, this setup can be used to generate any arbitrary vector beam by simply superimposing different scalar beams. For example, adding two vortex modes, $\hbox {LG}_0^1$ and $\hbox {LG}_0^{-1}$, will create radially and azimuthally polarized vortex beams.

#### Trapping with vector flat-top beams

**Figure 6:**
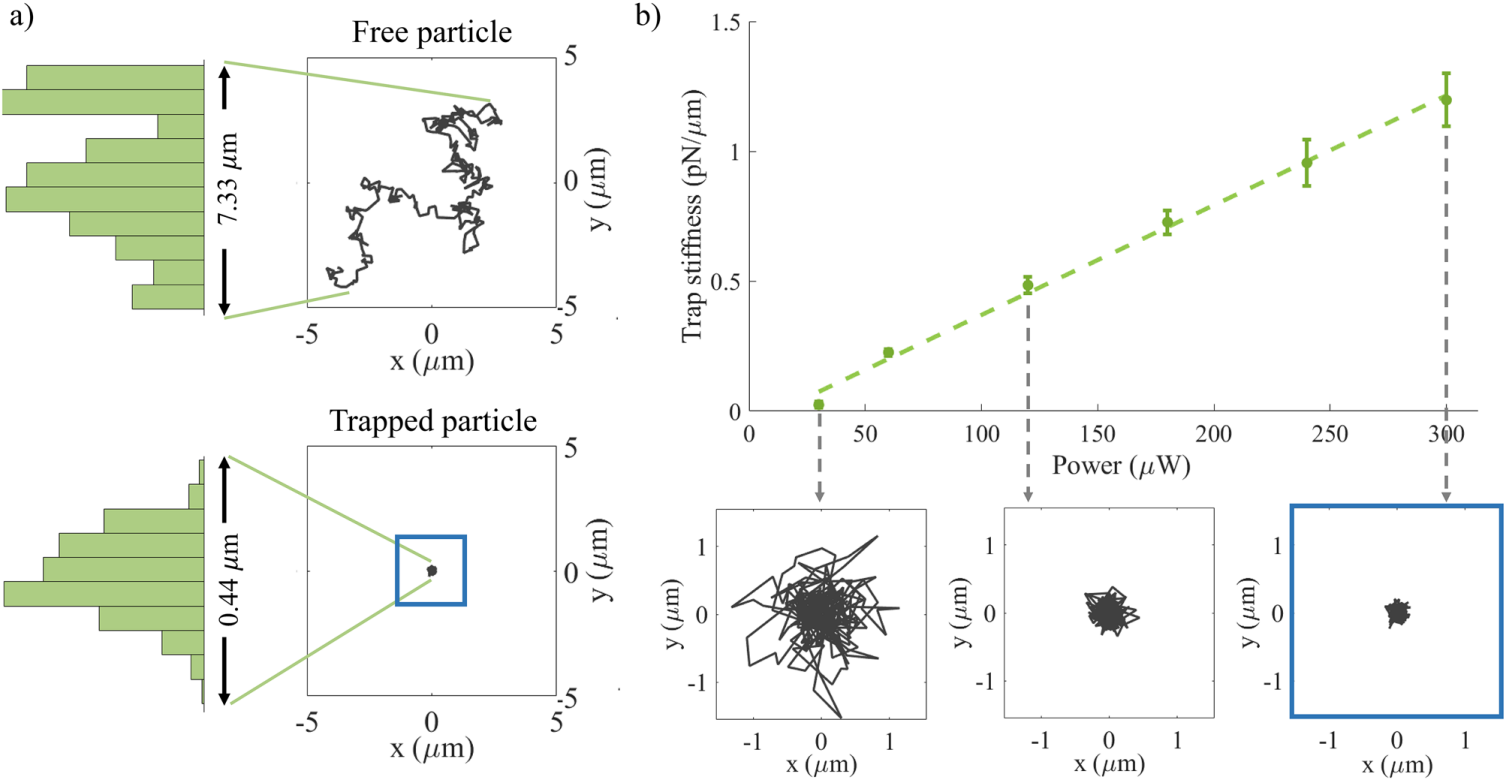
Optically trapping a 2 $\upmu \text{m}$ particle with a vector flat-top beam. (**a**) Trajectory and probability density (in the y-direction) of a free particle and a particle trapped with a vector flat-top beam, monitored for 5 min. The free particle exhibits Brownian motion and moves over 7.33 $\upmu \text{m}$ whereas the trapped particle’s movement is restricted to only 0.44 $\upmu \text{m}$. (**b**) Vector flat-top trap stiffness (in the x-direction) at different laser powers. The insets show the trajectory of a trapped particle at increasing powers of 30 $\upmu \text {W}$, 120 $\upmu \text {W}$ and 300 $\upmu \text {W}$, respectively.

The motion of a free particle and a particle trapped with a vector flat-top beam were monitored for 5 min. The trajectory and the distribution of these particles’ position in the Y-direction are shown in Fig. 6a. The particle position was tracked using the CCD camera and all image analysis was done in Matlab. From this figure, it is clear that the free particle underwent random Brownian motion and in 5 min moved over 7.33 $\upmu \text{m}$ (in the Y-direction). The trapped particle was, however, confined to move only 0.44 $\upmu \text{m}$ during the analysis time, which proves successful optical trapping with a vector flat-top beam.

The slight movement of the particle inside the trap is due to thermal noise pushing it out of the trap and the optical force drawing it back in. By monitoring this movement, the trap strength could be determined using the equipartition method which relates the trap strength to the position variance of the trapped particle^52^ (details given in the Supplementary Information). The motion of a trapped bead was tracked for 5 min, taking a position measurement every second. Five beads were trapped for each power measurement (measured after the objective lens) at which the trap stiffness was determined. The average trap stiffness at each power is plotted in Fig. 6b with the error bars being the standard error. The linear relationship between the laser power and the trap stiffness is evident from this graph. The insets show the trajectory of the trapped particle at increasing powers of 30 $\mu \hbox {W}$, 120 $\upmu \text {W}$ and 300 $\mu \hbox {W}$.

#### Tailoring the trap

**Figure 7:**
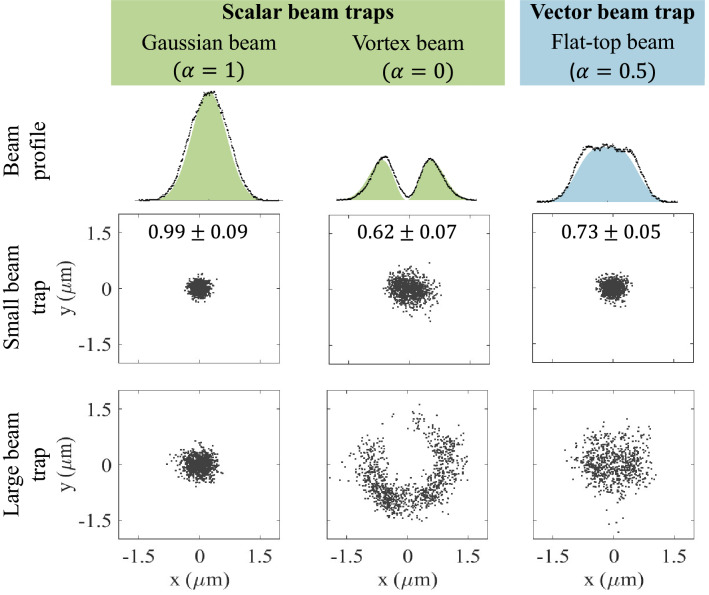
Movement of beads trapped with a Gaussian, vortex and vector flat-top beam when the beam size is smaller than the particle (small beam trap) or larger than the particle (large beam trap). The bead had a 2 $\upmu \text {m}$ diameter, the small trap had a beam diameter of 1.8 $\upmu \text{m}$ and the large trap a diameter of 3.1 $\upmu \text{m}$. The trap stiffness (± standard deviation) for the small beam traps are reported (unit: pN/$\upmu \text{m}$).

In Fig. 7 the tuneability of the vector HOT is demonstrated by changing the trapping beam from purely scalar Gaussian and vortex beams to their vectorial combination; here we also demonstrate the effect of trapping with different beam sizes. In the top row the theoretical profiles (plotted in color) of the trapping beams are shown and are in good agreement with the experimental data (presented in black).

The optical trapping force a particle will experience (due to a focused beam) depends on the beam size relative to the particle. In the theory presented earlier, the average gradient force of the beam was determined by integrating over the total area of the beam. The particle will, however, only experience this total force if it intercepts the entire beam; which is true when the beam at the trap is smaller than the particle size. This was the case for the trapped beads in the middle row of Fig. 7, here 2 $\upmu \text{m}$ beads were trapped with a beam diameter of 1.8 $\upmu \text{m}$. The bottom row shows the movement of a bead in an optical trap where the beam is larger (3.1 $\upmu \text{m}$) than the bead. The beam size was determined by imaging the back reflection of the beam from the sample slide. As mentioned previously, the dichroic mirrors are not 100% efficient, therefore the trapping beam was partly transmitted and imaged to the CCD camera. The camera was calibrated using the known diameter of the 2 $\upmu \text {m}$ beads in the sample. For the small traps, the movement of the bead was concentrated at the centre of all the beams with a little more movement in the flat-top beam and even more in the vortex beam. Harmonic oscillation could be assumed and the trap stiffness (calculated with the equipartition method) is reported for these traps. The movement of the bead in the large Gaussian trap was also centred but less stiff than for the small Gaussian trap. The centre of the particle trapped in the large vortex beam, stayed in the ring of the beam since this is where the intensity gradient and consequently the trapping force exist, movement along the ring of the beam was mainly due to Brownian motion. Lastly, the movement of the bead in the large flat-top trap was more uniform, corresponding to the uniform beam profile (only a gradient force exists at the edges of the beam). From these plots it is clear that the size and type of beam have a great influence on the strength and properties of an optical trap.

**Figure 8:**
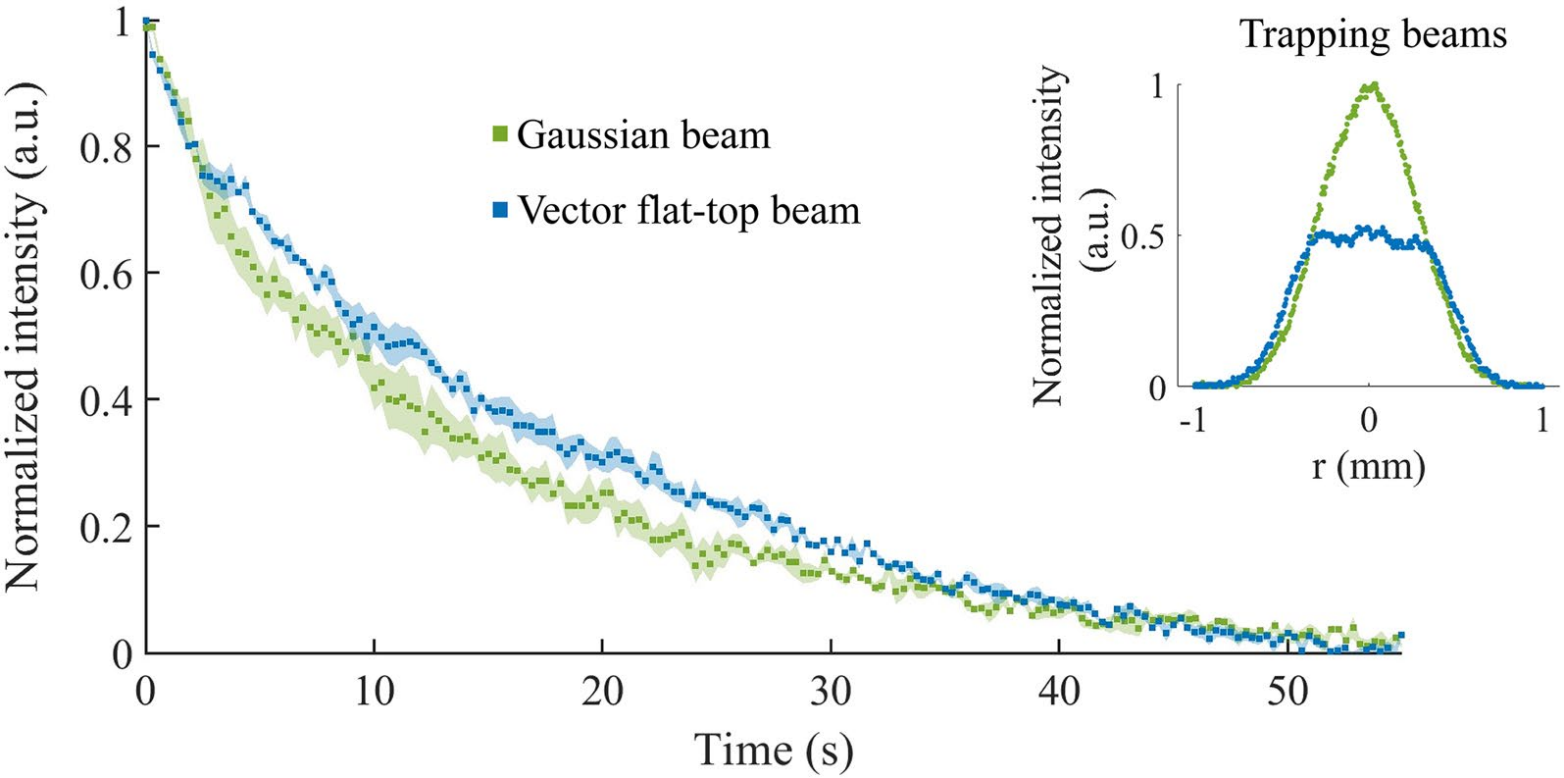
The average photobleaching of three trapped QD-tagged beads in a Gaussian and flat-top beam trap at a trapping power of 60 $\upmu \text {W}$ (the shaded area represents the standard error). Longer photobleaching lifetimes were observed for the fluorophores in the flat-top beam trap as compared to the Gaussian beam trap. The inset shows the experimental profiles of the trapping beams.

### Photobleaching effect

The vector flat-top beam can be generated to have the same gradient force as the Gaussian beam, but with a 25% reduced peak intensity (refer to Fig. 4). This property of flat-top beams can be exploited to reduce photobleaching in optical traps. Moreover, if flat-top beams are generated with the same power as Gaussian beams, they have a 50% reduced peak intensity, with only a minimal loss of gradient force (trap strength) especially at low trapping powers (as shown experimentally in Supplementary Fig. S3). Fig. 8 shows the average fluorescence emission of three QD-tagged beads trapped with a Gaussian or a flat-top beam, with a power of 60 $\mu \hbox {W}$ in a single wavelength trap (with the standard error represented by the shaded area). The fluorescence signals were normalized and background subtracted (detail in Supplementary Fig. S4). For both traps the fluorescence of the particle reduced upon entering the trap; the photobleaching half-life ($\tau$) of each particle was determined by fitting an exponential function $f(t) = A\hbox {exp}(-t/\tau )+C$ to the fluorescence signal (see Supplementary Fig S5 for fitted functions and $R^2$ values). The average photobleaching half-life (± standard deviation) of a QD-tagged bead in a Gaussian trap was determined to be 14 ± 3 s and the half-life in a vector flat-top trap 20 ± 3 s, which is a 43% increase in the photobleaching half-life.

## Discussion and conclusion

Although the application of optical trapping and tweezing in biophotonics and physics is very well established, its use in chemistry is far less developed. Here we introduce the full vectorial nature of light in enhancing light-based chemistry within a novel holographic trap using custom made QDs as our example. We point out that the combination of QD bead-based fluorescence assays and optical tweezers has been used for the detection of analytes such as prostate-specific antigen, the H5N1 and H7N9 avian influenza virus genes with high sensitivity and detection limits as low as 1.0–2.0 pM^53–55^. The QD probes synthesized in this work, therefore, have the potential to act as sensors inside the optical tweezer setup for the detection of analytes (like environmental pollutants^56–58^) with increased sensitivity, an exciting future prospect.

In conclusion, we have demonstrated an optical tweezer setup that uses vectorial light to trap and control QD fluorescent probes. We outlined the chemistry involved to functionalize micro-sized polymer spheres with QDs, highlighting the importance of size, adhesion and agglomeration control. The versatility of the vector HOT was demonstrated by switching between scalar and vector beams and trapping particles with different beam sizes. By trapping with a propagation invariant vector flat-top beam, we demonstrated the potential of reducing photobleaching in a single wavelength optical trap by simply tailoring the intensity gradient landscape.

## Materials and methods

### Chemicals

Cadmium oxide (CdO), octadec-1-ene (ODE), oleic acid (OA), trioctylphosphine oxide (TOPO), selenium (Se), zinc oxide (ZnO), sulfur (S), L-cysteine, N-(3-dimethylaminopropyl)-N’-ethylcarbodiimide hydrochloride (EDC), N-hydroxysuccinimide (NHS), methanol and acetone were purchased from Sigma Aldrich (USA). Chloroform, ethanol and potassium hydroxide (KOH) were purchased from Associated Chemical Enterprises (South Africa). Argon gas baseline 5.0 from Afrox (South Africa) was used. Deionized water used during the syntheses was from an in-house Drawell Eco-Q deionized water system (China). $\hbox {Invitrogen}^{\text {TM}}$ 2 $\mu$m carboxyl functionalized latex beads were purchased from Thermo Fisher Scientific (South Africa).

### Quantum dot synthesis

Firstly, selenium (0.30 g Se, 1.94 g TOPO and 25 ml ODE), zinc (0.21 g ZnO, 10 ml OA and 15 ml ODE) and sulphur (0.087 g S, 10 ml OA and 15 ml ODE) precursor solutions were prepared. These solutions were stirred at 40 $^{\circ }\hbox {C}$ for 5 hr to ensure thorough mixing. The QD synthesis setup included a three-necked round bottom flask fitted with a condenser, thermometer and an argon gas inlet positioned on a heating mantle. The whole reaction was done under argon conditions. 1.3 g CdO, 50 ml ODE and 30 ml OA were added to the flask and stirred vigorously at 260 $^{\circ }\hbox {C}$ until a colourless solution formed indicating the formation of the Cd-OA complex. The Se-precursor was added to the flask (25 ml) and nucleation and core growth were allowed to proceed for 15 min at a temperature of $\sim 240$ $^{\circ }\hbox {C}$. The epitaxial ZnS shell growth around the core was initiated by adding 10 ml of the Zn-precursor, and swiftly thereafter 10 ml of the S-precursor to the core solution. Shell growth was left to proceed for 40 min at a temperature of 240 $^{\circ }\hbox {C}$. After 40 min the reaction was cooled to room temperature. The QDs were purified with methanol by centrifugation which then yielded the hydrophobic CdSe/ZnS QDs capped with OA and TOPO.

A ligand-exchange reaction was carried out next to functionalize the surface of the CdSe/ZnS QDs with L-cysteine in order to make them hydrophilic. A solution of 4.4 g KOH, 60 ml MeOH and 3 g L-cysteine was prepared and placed in an ultrasonic bath for 10 min to ensure all the L-cysteine dissolved. The hydrophobic CdSe/ZnS QD solution was suspended in chloroform and added to the L-cysteine solution. Deionized water was slowly added to the mixture while stirring at room temperature which changed the transparent orange solution to milky. After an hour of stirring, the solution was left to stand overnight to ensure complete separation of the organic and aqueous phases. The L-cysteine capped QDs, now in the aqueous phase, were purified by centrifugation with ethanol ($\times 4$) and acetone ($\times 2$). Rigorous purification was necessary to remove the excess organic compounds from the surface of the QDs to achieve a monodispersed QD solution with no agglomerates. The absorbance and FTIR spectra of the QD-products are shown in Supplementary Fig. S6 and S7, respectively.

### Coupling reaction

To couple the L-cysteine-capped QDs to micro-sized polymer beads, 2.5 ml of EDC (0.1 M) and 2.5 ml NHS (0.1 M) were added to 50 $\mu \hbox {l}$ of the polymer beads (diluted in 1 ml water) and stirred for 30 min in an ice bath to activate the carboxylic acid groups on the beads. Excess EDC was removed by centrifugation with deionized water. After centrifuging, the activated beads were redispensed in 4 ml water and 3 mg of QDs was added. The coupling reaction was left to proceed in an ultrasonic bath for 2 h to ensure even coating of the QDs on the beads. The coupled product was purified by centrifugation with water ($\times 3$) and stored in deionized water in the fridge. The FTIR spectra of the QD-tagged beads are shown in Supplementary Fig. S8.

## Supplementary Information

Supplementary Information.

## Data Availability

The datasets used and/or analyzed during the current study are available from the corresponding author upon reasonable request.
