# Supplementary material for: Optical trapping and fluorescence control with vectorial structured light
Source: Sci Rep. 2022 Oct 21;12:17690. doi: 10.1038/s41598-022-21224-1 (PMC9587264; doi:10.1038/s41598-022-21224-1)
Supplement: Supplementary file 1 — Supplementary Information. [file 41598_2022_21224_MOESM1_ESM.pdf]

Supplementary information

Optical trapping and fluorescence control with vectorial structured light

Ané Kritzinger,[a] Andrew Forbes[b] and Patricia B.C. Forbes[a*]

[a] Department of Chemistry, University of Pretoria, Pretoria, South Africa
[b] School of Physics, University of the Witwatersrand, Johannesburg, South Africa
∗ Corresponding author email: patricia.forbes@up.ac.za

Effect of the dichroic mirror

The reflection of horizontally and vertically polarized light from the dichroic mirror (DM) in the optical tweezer is slightly different. As mentioned in the main text, to minimize the effect on the profile of the beam entering the trap, a quarter wave plate (QWP) at 45° was added in the path of the vector beam to change the polarization of each beam to circular (meaning the two beams making the vector light have the same 'amount' of vertical and horizontal polarization). Fig. S1 shows the profile of a flat-top beam before the DM as well as after reflecting from the DM with and without a QWP added in the setup, confirming improvement of the beam profile upon adding a QWP.

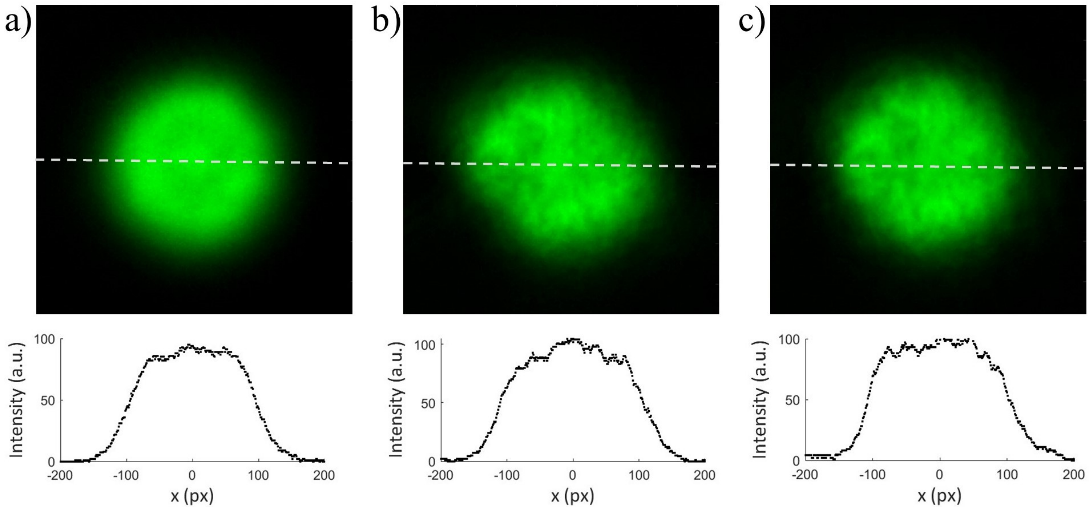

Figure S1: The beam profile and cross-section of a flat-top beam a) before the dichroic mirror (DM) and after reflection from the DM b) without a QWP and c) with a QWP added in the setup.

Trap calibration: the equipartition method

An optical trap can be described as a microscopic spring. The particle is kept in the equilibrium position by a harmonic force defined by Hooke's law[1]

$$F_{OT} = -\kappa_x(x - x_{eq}), \tag{S1}$$

where F_{OT} is the applied force by the optical trap, κ_x is called the trap stiffness in the x-direction and $(x - x_{eq})$ the displacement of the particle from the equilibrium position (x_{eq}). The force in the y-direction can be obtained by considering the displacement in this direction.

The resulting trapping potential is given by

$$U(x) = \frac{1}{2}\kappa_x \left(x - x_{eq}\right)^2 \ . \tag{S2}$$

The equipartition theorem can then be used to measure the trap stiffness. This theorem states that the energy of a system at equilibrium is $\frac{1}{2}k_BT$ for each degree of freedom, with k_B Boltzmann's constant and T the temperature at equilibrium. Therefore,

$$\langle U\left(x\right)\rangle = \frac{1}{2}\kappa_x \langle \left(x - x_{eq}\right)^2\rangle = \frac{1}{2}k_BT \, . \tag{S3}$$

Let $\sigma_x^2 = \langle \left(x - x_{eq}\right)^2\rangle$, the variance of the position of the trapped particle, then

$$\kappa_x = \frac{k_BT}{\sigma_x^2} \, . \tag{S4}$$

Thus by measuring the position variance, the trap stiffness was be determined. The calculation of σ_x^2 is straightforward, but requires calibrated position detection which was done using a CCD camera. The position of the particle is needed in units of meters, but since the camera gave the displacement in pixels a conversion is needed. The pixel size of the camera was determined by using an object of known dimensions. The polystyrene sphere with a known diameter of 2 μm was used and the size of a pixel was determined to be 53.3 nm. A temperature of 22 °C and $k_B = 1.3806 \times 10^{-23}$ m^{2}kg/s^{2}K were used for the trap stiffness calculations.

Fig. S2a shows the x-displacement of the particle in a trap over 5 min. The equilibrium position is at $x = 0$. Fig. S2b shows the corresponding position distribution with a fitted Gaussian curve and S2c the trap potential in units of k_BT with a fitted parabolic function, confirming harmonic potential. In Fig. S2d the the potential wells of optical traps at powers of 30 μW, 120 μW and 300 μW are shown - the wells became narrower as the power or trap stiffness increased.

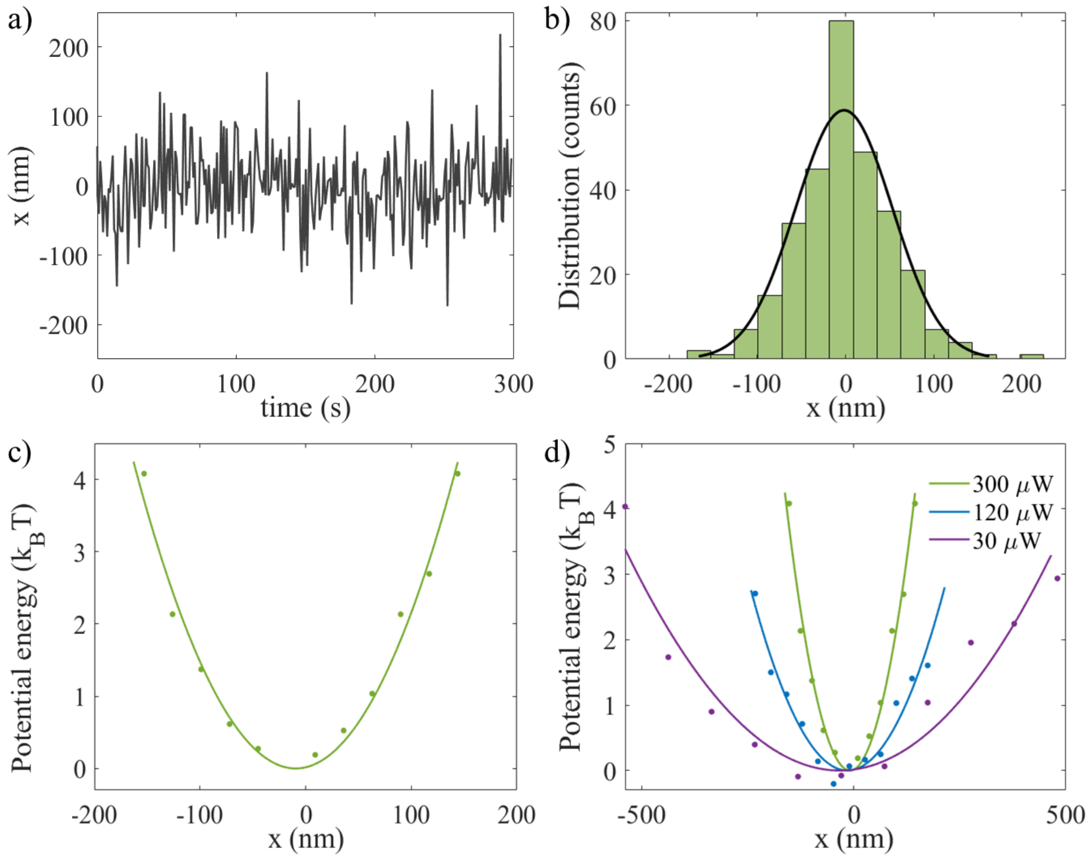

Figure S2: Potential analysis of a trapped particle. a) The displacement of the particle centre from the equilibrium position over time and b) the corresponding position distribution and c) potential energy of the trap. d) Comparison of optical trap potential wells at increasing powers of 30 μW, 120 μW and 300 μW.

Comparing flat-top and Gaussian beam traps

From Fig. 4 (main text) we concluded that at the same power, the Gaussian beam will (theoretically) create a stronger optical trap compared to the flat-top beam due to its high peak intensity. This was confirmed experimentally by determining the trap stiffness of these two beams at different powers. Five 2 μm diameter beads were trapped (for 3 min) for each power measurement with both the Gaussian and the flat-top beam. The average trap stiffness of the five beads is plotted in Fig. S3 with the standard error shown by the error bars. From this plot it is clear that the Gaussian beam outperformed the flat-top beam at each power measurement, however, at lower powers the difference in trap stiffness became smaller.

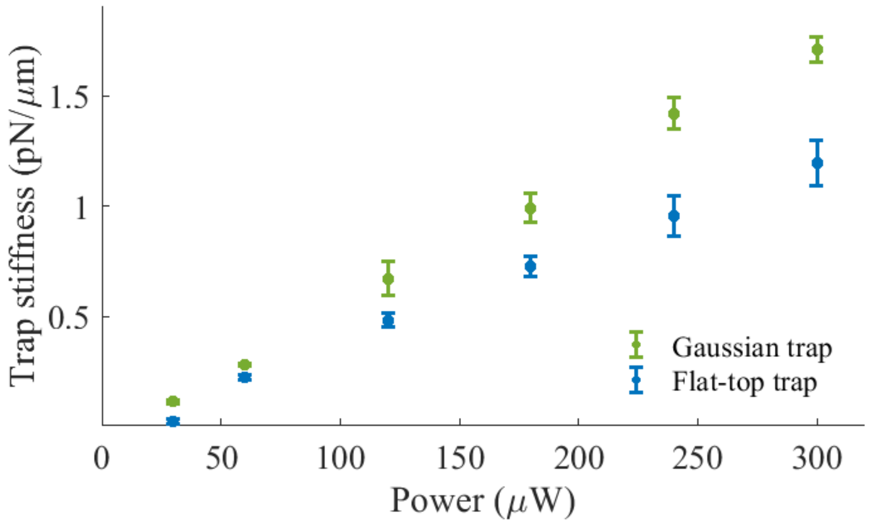

Figure S3: The trap stiffness of a Gaussian and flat-top beam at different powers.

Fluorescence signal from the QD-tagged bead sample

The fluorescence signal from the QD-tagged bead sample, detected with an APD, is shown in Fig. S4 (all measurements were taken with an integration time of 300 ms). The background noise from the QD-sample ($\sim$ 2700 counts), shown at times 0-32 s, is due to the presence of unreacted QDs that did not couple to the bead. The fluorescence emission of an optically trapped QD-tagged bead is shown at times 32 - 180 s. The QD-tagged bead experienced photobleaching inside the trap, and after $\sim$ 90 s it was completely bleached. The background signal was subtracted for the results presented in the main text (Fig. 8).

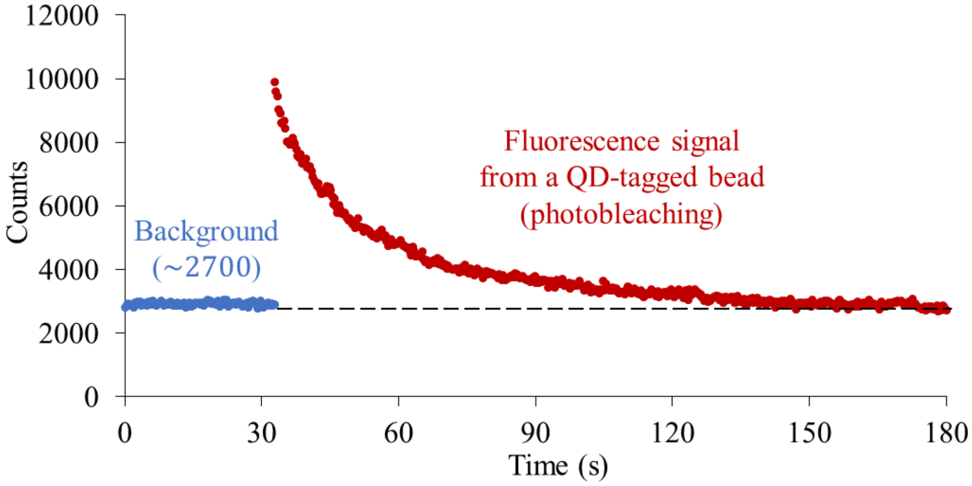

Figure S4: The fluorescence emission of an optically trapped QD-tagged bead detected with an APD (times 32 - 180 s) with the background counts shown at times 0 - 32 s.

Exponential fits to photobleaching curves

Fig. S5 shows the the photobleaching decay of QD-tagged beams in a Gaussian or vector flat-top beam trap. The bleaching half-life (τ) of each particle was determined by fitting an exponential function $f(t) = A\exp(-t/\tau) + C$ to the fluorescence signal; the half-life and R^2 for each fit is reported in the graph.

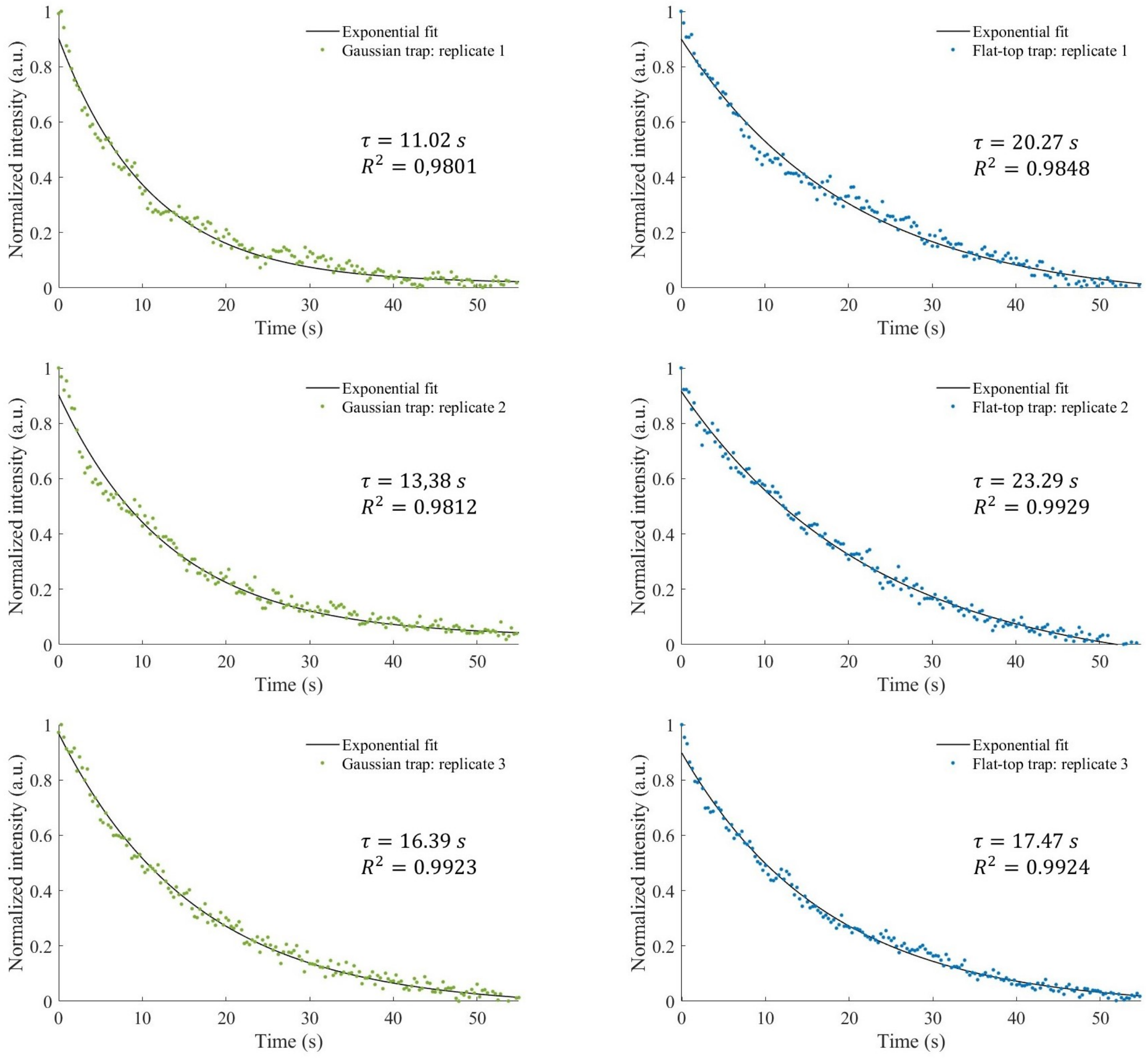

Figure S5: Exponential fits to the photobleaching decays of a QD-tagged bead (3 replicates) in a Gaussian beam (green) or a flat-top beam (blue) trap. The photobleaching half-life τ for each bead is reported.

QD and QD-tagged bead characterization

UV-vis absorbance

The absorbance spectrum of the L-cysteine capped QDs is shown in Fig. S6. The broad absorbance spectrum is characteristic of QDs. The two absorption wavelengths indicated on the spectrum were of importance during this study. Firstly, 470 nm was the excitation wavelength used for fluorescence emission monitoring during synthesis and for characterization purposes. The second important wavelength was 532 nm since this was the wavelength of the laser used for excitation in the optical tweezer setup.

Quantum yield

The comparative method was used to determine the quantum yield (Φ_{QD}) of the QDs using rhodamine 6G (Rh) as the reference.[2,3]

$$\Phi_{\text{QD}} = \Phi_{\text{std}} \cdot \left(\frac{F_{\text{QD}}}{F_{std}}\right) \cdot \left(\frac{A_{std}}{A_{\text{QD}}}\right) \cdot \left(\frac{n_{QD}^2}{n_{std}^2}\right),$$

where F is the integrated fluorescence intensity, A the absorbance at the excitation wavelength and n the refractive index of the solvent used for the standard Rh sample (std) and the QD sample. Using the

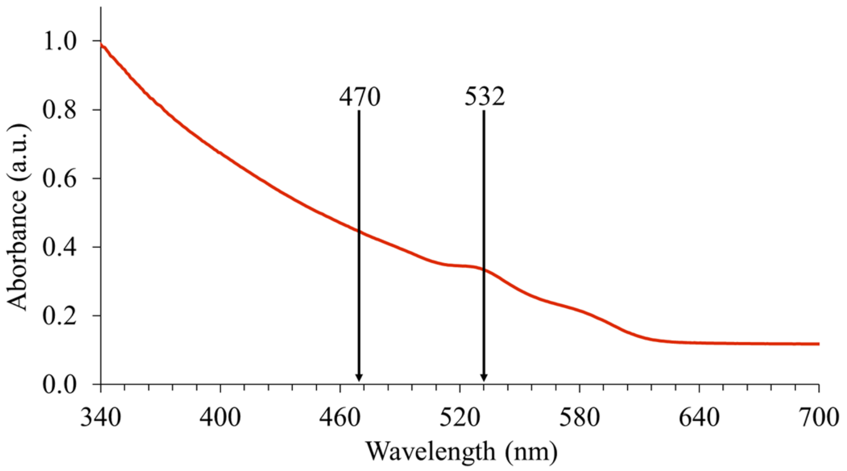

Figure S6: UV-vis absorbance of L-cysteine capped CdSe/ZnS QDs in water.

absolute quantum yield of Rh in water ($\Phi_{\text{std}} = 0.90$)[4] and $n_{QD} = n_{std} = 1.333$, the QY of the L-cysteine capped CdSe/ZnS QDs was determined to be **48%**.

FTIR analysis

In order to determine the surface chemistry of the QDs and to confirm the success of the ligand exchange reaction, FTIR spectra of the hydrophobic QDs, L-cysteine capped QDs, pure TOPO and L-cysteine were measured – see Fig. S7. All the spectra and peak positions correspond well to reported literature.[5–10]

The spectrum of TOPO shows peaks at 2918 and 2849 cm^{-1} corresponding to the symmetric and asymmetric stretching of the CH_2 groups in the alkyl chains. These peaks are also seen in the spectra of the TOPO capped hydrophobic QDs. The characteristic $P=O$ and $P-C$ stretching of pure TOPO were observed at 1145 and 1464 cm^{-1}, respectively. The absence of the $P=O$ peak in the hydrophobic QDs confirms the coordination of TOPO to the QD surface. The peaks at 1530 and 1434 cm^{-1} in the hydrophobic QD spectra can be ascribed to the $P-C$ stretching of coordinated TOPO.[7]

The $C=O$ and $C-O$ stretches of the carboxylic group in L-cysteine are seen around 1550 and 1390 cm^{-1} in the L-cysteine capped QDs and the broad peak ~3200 cm^{-1} is from the $O-H$ stretch. The $S-H$ vibration (2550 - 2750 cm^{-1}) of L-cysteine disappeared in the spectra of the L-cysteine capped QDs confirming covalent bond formation between L-cysteine and ZnS for the CdSe/ZnS QDs and the formation of $Cd-S$ in the water-soluble CdSe core QDs.[5] The FTIR analysis verified (along with the other characterization techniques) the successful synthesis of L-cysteine capped CdSe/ZnS QDs.

Fig. S8 compares the FTIR spectra of the two starting materials (L-cysteine capped QDs and the polystyrene beads) with the QD-tagged bead product. The spectrum of the product is dominated by the beads with the addition of two new peaks at 1733 and 1220 cm^{-1}. These peaks can be ascribed to the amide linkage formed between the carboxylic acid on the beads and the L-cysteine on the QDs with the $C=O$ stretch corresponding to 1733 cm^{-1} and $C-N$ to 1220 cm^{-1}.[11–13]

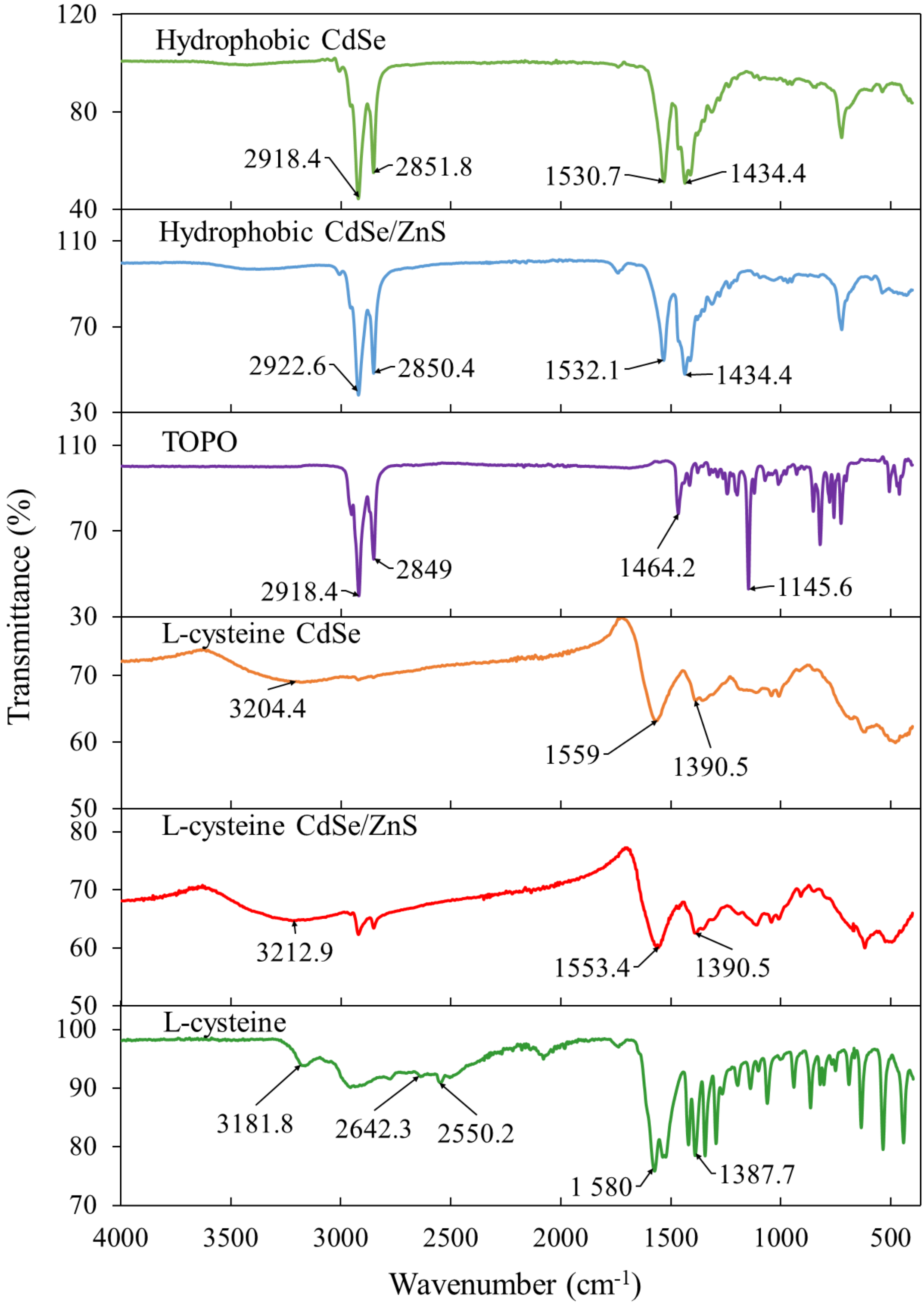

Figure S7: FTIR spectra of the hydrophobic and L-cysteine capped core and core/shell QDs as well as the spectra of TOPO and L-cysteine.

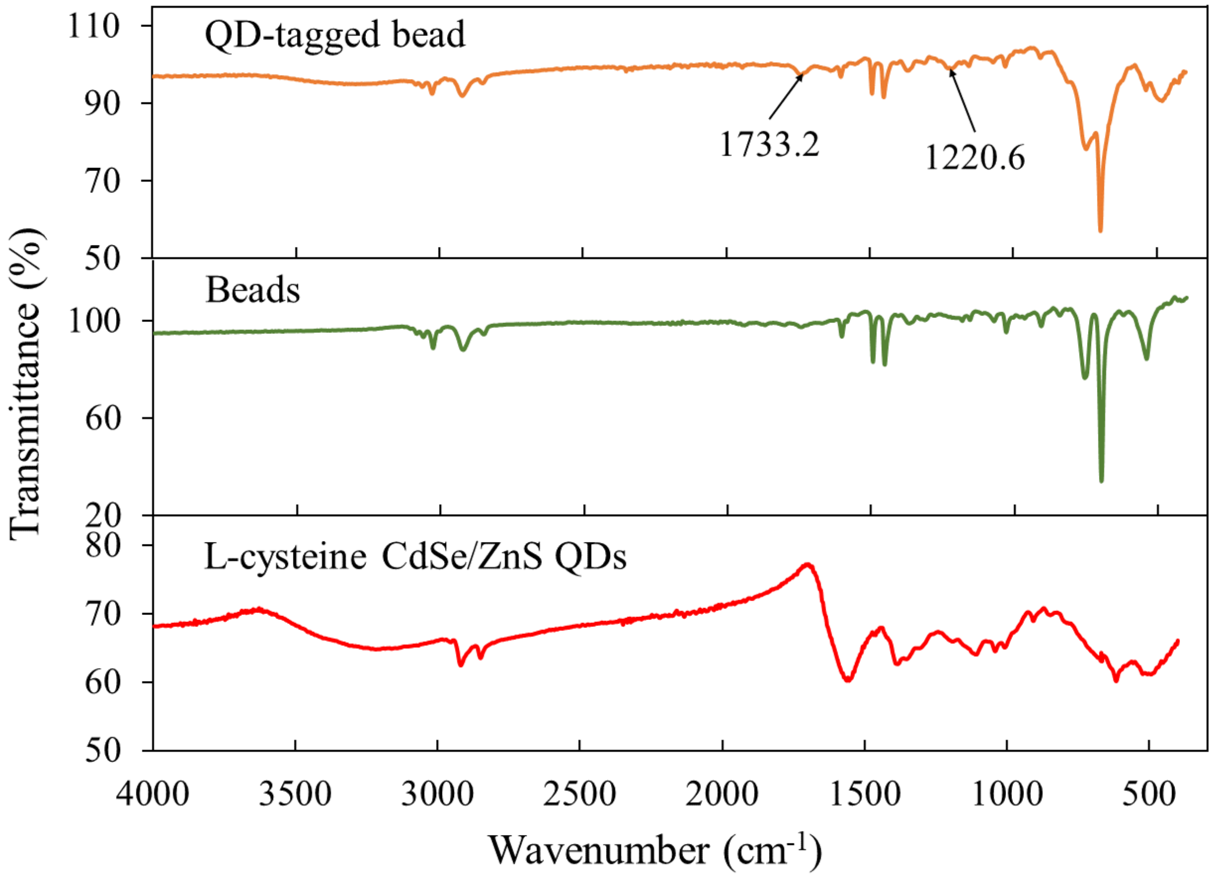

Figure S8: FTIR spectra of the QD-tagged beads, uncoated commercial beads and L-cysteine capped QDs.

References

[1] J. Gieseler, J. R. Gomez-Solano, A. Magazzù, I. Pérez Castillo, L. Pérez García, M. Gironella-Torrent, X. Viader-Godoy, F. Ritort, G. Pesce, A. V. Arzola, K. Volke-Sepúlveda and G. Volpe, *Advances in Optics and Photonics*, 2021, **13**, 74–241.

[2] A. T. Williams, S. A. Winfield and J. N. Miller, *Analyst*, 1983, **108**, 1067–1071.

[3] S. Fery-Forgues and D. Lavabre, *Journal of Chemical Education*, 1999, **76**, 1260.

[4] D. Magde, R. Wong and P. G. Seybold, *Photochemistry and Photobiology*, 2002, **75**, 327–334.

[5] H. Montaseri and P. B. Forbes, *Materials Today Communications*, 2018, **17**, 480–492.

[6] H. Montaseri, O. Adegoke and P. B. Forbes, *South African Journal of Chemistry*, 2019, **72**, 108–117.

[7] X. Nie, Y. Zhang, X. Wang, C. Ren, S.-Q. Gao and Y.-W. Lin, *ChemistrySelect*, 2018, **3**, 2267–2271.

[8] S. N. Inamdar, P. P. Ingole and S. K. Haram, *ChemPhysChem*, 2008, **9**, 2574–2579.

[9] S. A. Nsibande and P. B. Forbes, *Luminescence*, 2019, **34**, 480–488.

[10] V. V. Doan-Nguyen, P. J. Carroll and C. B. Murray, *Acta Crystallographica Section C: Structural Chemistry*, 2015, **71**, 239–241.

[11] Y. Ji, X. Yang, Z. Ji, L. Zhu, N. Ma, D. Chen, X. Jia, J. Tang and Y. Cao, *ACS Omega*, 2020, **5**, 8572–8578.

[12] O. Adegoke and P. B. Forbes, *Talanta*, 2016, **146**, 780–788.

[13] J. Coates, in *Encyclopedia of Analytical Chemistry*, ed. R. A. Meyers, John Wiley & Sons, 200, pp. 10815–10837.
